# Inpatient Psychiatric Unit Availability Within US Short-Term, Acute-Care Hospitals, 2011-2023

**DOI:** 10.1001/jamanetworkopen.2025.18881

**Published:** 2025-06-30

**Authors:** Zoe Lindenfeld, Colleen M. McCullough, Ji Eun Chang, Jonathan H. Cantor, Ryan K. McBain

**Affiliations:** 1 Edward J. Bloustein School of Planning and Public Policy, Rutgers University, New Brunswick, New Jersey; 2 RAND, Santa Monica, California; 3 Department of Public Health Policy and Management, School of Global Public Health, New York University, New York, New York

## Abstract

This cross-sectional study examines the availability of inpatient psychiatric units in short-term, acute-care hospitals from 2011 to 2023.

## Introduction

The COVID-19 pandemic caused disruptions to behavioral health care delivery, despite increased demand for behavioral health services. This involved closing inpatient psychiatric units (IPUs), hospital-based units that provide psychiatric services to patients with mental illness, or repurposing them to increase medical capacity.^1^ This study examines the availability of IPUs in short-term, acute-care hospitals from 2011 to 2023. We quantify changes in IPUs over time and measure the association of the COVID-19 pandemic with the number of IPUs.

## Methods

This cross-sectional study followed the STROBE reporting guideline. The study was deemed exempt by the institutional review board by Rutgers University per institutional policy. We used data from the 2011 to 2023 US Centers for Medicaid & Medicare Services Medicare hospital cost reports.^2^ All Medicare-certified hospitals are required to complete a cost report each year. We extracted year data on hospital type, IPUs, and urbanicity. Our sample included general short-term, acute-care hospitals that indicated presence or absence of an IPU on the cost report. A flow diagram for the creation of the analytic sample is provided in the eMethods in Supplement 1.

We assessed the losses and gains of IPUs from 2011 to 2023, and we calculated the percentage of hospitals with an IPU among short-term, acute-care hospitals during the indicated year. Following the approach of prior research,^3^ we estimated ordinary least-squares regression in the prepandemic period (2011 to 2019) and estimated the number of IPUs in the pandemic period (2020 to 2023), quantifying what IPU availability would have been in the absence of the pandemic, assuming prepandemic trends continued. We calculated percentage differences between actual and estimated IPU availability and used mean comparison tests to compare these values. As a sensitivity analysis, we quantified comparative changes in intensive care units (ICUs) during the same period. Analyses were conducted with Stata version 18.0 (StataCorp); *P* < .05 defined statistical significance, and all tests were 2-sided.

## Results

Our final sample included 5017 unique hospitals that completed at least 1 cost report between 2011 and 2023, 1985 located in rural counties (39.6%) and 60.4% in urban counties (3032). In 2011, 24.9% (1156) of hospitals had IPUs, and there was a net loss of IPUs within hospitals in each subsequent year, with the exception of 2013 (Table). The percentage of hospitals with an IPU shrank to 20.6% (903) in 2023.

**Table.** Changes in Inpatient Psychiatric Unit Availability at US Short-Term, Acute-Care Hospitals, 2011 to 2023^a^

Year	Short-Term, Acute-Care Hospitals			
	Total, No.	With inpatient psychiatric units, No. (%)	Gained inpatient psychiatric units from previous year, No. (%)	Lost inpatient psychiatric units from previous year, No. (%)
2011	4646	1156 (24.8)	NA	NA
2012	4630	1142 (24.6)	32 (0.69)	46 (0.99)
2013	4646	1142 (24.5)	32 (0.69)	32 (0.69)
2014	4629	1140 (24.6)	35 (0.76)	37 (0.80)
2015	4591	1117 (24.3)	23 (0.50)	46 (1.00)
2016	4559	1113 (24.4)	26 (0.57)	30 (0.66)
2017	4531	1100 (24.2)	22 (0.49)	35 (0.77)
2018	4513	1068 (23.6)	18 (0.40)	50 (1.11)
2019	4480	1040 (23.2)	24 (0.54)	52 (1.16)
2020	4452	1004 (22.5)	19 (0.43)	55 (1.24)
2021	4431	967 (21.8)	11 (0.25)	48 (1.08)
2022	4421	942 (21.3)	12 (0.27)	37 (0.84)
2023	4393	903 (20.5)	9 (0.20)	48 (1.09)

Abbreviation: NA, not applicable.

^a^ Data from the 2011 to 2023 Centers for Medicare and Medicaid Services Hospital Cost Report Information System were analyzed.

In the prepandemic period, the number of IPUs decreased less rapidly (−14.11; 95% CI, −18.28 to −9.95; *P* < .01) compared with the pandemic period (−35.1; 95% CI, 46.54 to −23.65; *P* < .01). The percentage differences in observed vs estimated IPUs were −7.90 (95% CI, −12.46 to −3.33; *P* < .05) for all hospitals, −11.34 (95% CI, −18.70 to −3.98; *P* < .05) for hospitals in rural counties, and −6.75 (95% CI, −10.39 to −3.12; *P* < .01) for hospitals in urban counties (Figure). We failed to observe a similar decline in ICUs during the same period (Figure).

**Figure.**
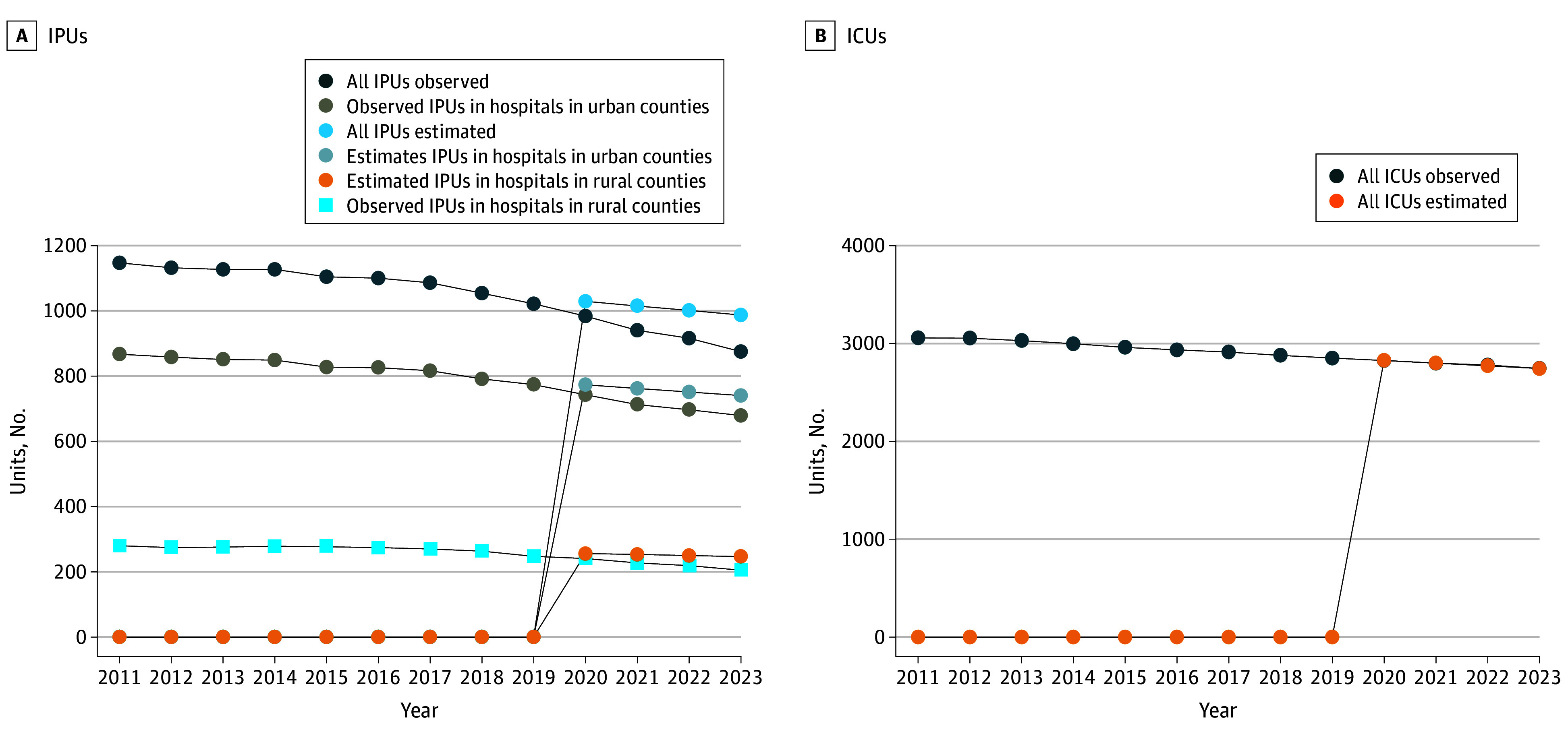
Actual vs Estimated Trends in Inpatient Psychiatric Units (IPUs) and Intensive Care Units (ICUs) in the US Data from the 2011 to 2023 the US Centers for Medicare & Medicaid Services Hospital Cost Report Information System were analyzed. The estimated number of IPUs and ICUs after the onset of the COVID-19 pandemic was estimated using ordinary least-squares regression.

## Discussion

The number of IPUs within short-term, acute-care hospitals has been decreasing for more than a decade^4^ and accelerated during the COVID-19 pandemic with a disproportionate decline in hospitals located in rural counties—nearly twice that of hospitals located in urban counties. This is concerning as IPUs provide critical evaluation, medication, and stabilization services, particularly in low-resourced and rural communities that may lack other behavioral health clinicians.^5^

Study limitations include the inability to assess how IPU changes affected patient outcomes and that causality cannot be attributed to the COVID-19 pandemic. For example, 1 explanation for our findings is that psychiatric services often offer lower reimbursement rates.^6^ As a result, hospitals may be reluctant to convert medical surgical beds, which generate higher revenue, into psychiatric beds.^6^ Future research should identify possible causes for the ongoing reduction in IPUs and measure how changes in IPU availability impacts public and behavioral health outcomes, including suicide and overdose.

## Notes

Supplement 1. **eFigure.** Consort Diagram Demonstrating the Creation of the Analytic Sample

Supplement 2.
Data Sharing Statement

## References

[1] Berezin J, Casoy F, Erlich MD, Hernandez Y, Smith TE. Inpatient psychiatry during COVID-19: a systems perspective. Psychiatr Clin North Am. 2022;45(1):45-55. 35219441 10.1016/j.psc.2021.11.002 PMC8580853

[2] RAND Hospital Data. RAND Corporation. Accessed May 27, 2025. https://www.rand.org/pubs/tools/TL303.html

[3] Nguyen T, Whaley C, Simon KI, Cantor J. Changes in employment in the US health care workforce, 2016-2022. JAMA. 2023;330(20):2018-2019. 37917055 10.1001/jama.2023.18932 PMC10623300

[4] McBain RK, Cantor JH, Eberhart NK. Estimating psychiatric bed shortages in the US. JAMA Psychiatry. 2022;79(4):279-280. 35171208 10.1001/jamapsychiatry.2021.4462

[5] Shumway M, Alvidrez J, Leary M, . Impact of capacity reductions in acute public-sector inpatient psychiatric services. Psychiatr Serv. 2012;63(2):135-141. 22302330 10.1176/appi.ps.201000145

[6] NY state senate and assembly joint FY2022 budget hearing: mental hygiene. New York State Nurses Association. Accessed May 27, 2025. https://nyassembly.gov/write/upload/publichearing/001184/002884.pdf

