# Supplementary material for: Inpatient Psychiatric Unit Availability Within US Short-Term, Acute-Care Hospitals, 2011-2023
Source: JAMA Netw Open. 2025 Jun 30;8(6):e2518881. doi: 10.1001/jamanetworkopen.2025.18881 (PMC12210080; doi:10.1001/jamanetworkopen.2025.18881)
Supplement: Supplement 1. — eFigure. Consort Diagram Demonstrating the Creation of the Analytic Sample [file jamanetwopen-e2518881-s001.pdf]

Supplemental Online Content

Lindenfeld Z, McCullough CM, Chang JE, Cantor JH, McBain RK. Inpatient psychiatric unit availability within US short-term acute care hospitals, 2011-2023. *JAMA Netw Open*. 2025;8(6):e2518881. doi:10.1001/jamanetworkopen.2025.18881

eFigure. Consort Diagram Demonstrating the Creation of the Analytic Sample

This supplemental material has been provided by the authors to give readers additional information about their work.

© 2025 Lindenfeld Z et al. *JAMA Network Open*.

eFigure. Consort Diagram Demonstrating the Creation of the Analytic Sample

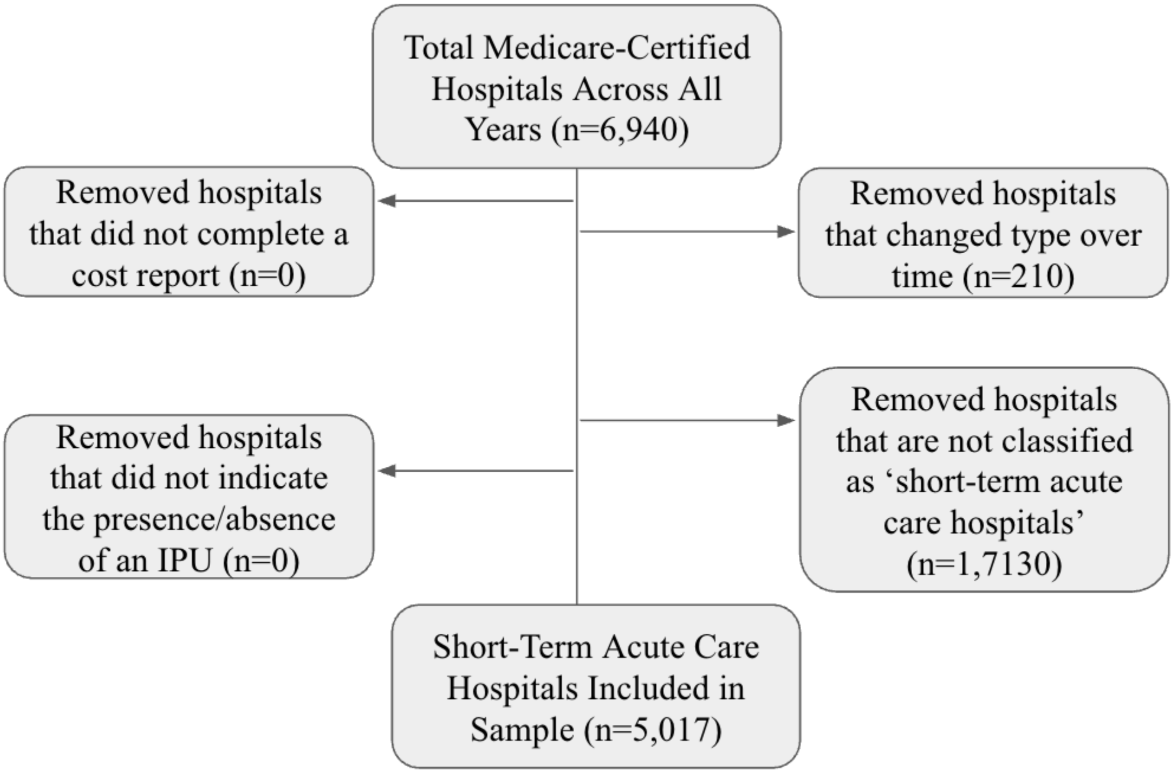

© 2025 Lindenfeld Z et al. *JAMA Network Open*.
