# Supplementary material for: Inpatient Psychiatric Unit Availability Within US Short-Term, Acute-Care Hospitals, 2011-2023
Source: JAMA Netw Open. 2025 Jun 30;8(6):e2518881. doi: 10.1001/jamanetworkopen.2025.18881 (PMC12210080; doi:10.1001/jamanetworkopen.2025.18881)
Supplement: Supplement 2. — Data Sharing Statement [file jamanetwopen-e2518881-s002.pdf]

Data Sharing Statement

Data

Data available: No

Additional Information

Explanation for why data not available: This study uses publicly available data.
